# Intensity-modulated versus 3-dimensional conformal radiotherapy in the definitive treatment of esophageal cancer: comparison of outcomes and acute toxicity

**DOI:** 10.1186/s13014-017-0863-3

**Published:** 2017-08-15

**Authors:** Matthias Felix Haefner, Kristin Lang, Vivek Verma, Stefan Alexander Koerber, Lorenz Uhlmann, Juergen Debus, Florian Sterzing

**Affiliations:** 1 Department of Radiation Oncology, University Hospital of Heidelberg, Im Neuenheimer Feld 400, 69120 Heidelberg, Germany; 2 National Center for Radiation Research in Oncology (NCRO), Heidelberg Institute for Radiation Oncology (HIRO), Im Neuenheimer Feld 400, 69120 Heidelberg, Germany; 3 Department of Radiation Oncology, University of Nebraska Medical Center, Omaha, NE USA; 4 Institute of Medical Biometry and Informatics (IMBI), University of Heidelberg, Im Neuenheimer Feld 130.3, 69120 Heidelberg, Germany; 5 Department of Radiation Oncology, Hospital Kempten, Robert-Weixler-Strasse 50, 87439, Kempten, Germany

**Keywords:** Esophageal cancer, Chemoradiotherapy, Toxicity, Radiation therapy, Intensity-modulated radiotherapy, Three-dimensional conformal radiotherapy

## Abstract

**Background:**

Though the vast majority of seminal trials for locally advanced esophageal cancer (EC) utilized three-dimensional conformal radiotherapy (3DCRT), the advanced and highly conformal technology known as intensity-modulated radiotherapy (IMRT) can decrease doses to critical cardiopulmonary organs. To date, there have been no studies comparing both modalities as part of definitive chemoradiation (dCRT) for EC. Herein, we investigated local control and survival and evaluated clinical factors associated with these endpoints between cohorts.

**Methods:**

We retrospectively analyzed 93 patients (3DCRT *n* = 49, IMRT *n* = 44) who received dCRT at our institution between 2000 and 2012 with the histologic diagnosis of nonmetastatic EC, a Karnofsky performance status of ≥70, curative treatment intent, and receipt of concomitant CRT. Patients were excluded if receiving <50 Gy. Kaplan-Meier analysis was used to evaluate the endpoints of local relapse rate (LR), progression-free survival (PFS), and overall survival (OS). Cox proportional hazards modeling addressed factors associated with outcomes with univariate and multivariate approaches. Rates of acute toxicities and basic dosimetric parameters were compared between 3DCRT and IMRT patients.

**Results:**

Mean follow-up was 34.7 months. The 3-year LR was 28.6% in the 3DCRT group and 22.7% in the IMRT group (*p* = 0.620). Median PFS were 13.8 and 16.6 months, respectively (*p* = 0.448). Median OS were 18.4 and 42.0 months, respectively (*p* = 0.198). On univariate analysis, only cumulative radiation dose was associated with superior LR (hazard ratio (HR) 0.736; 95% confidence interval (CI) 0.635 – 0.916, *p* = 0.004). Factors clearly affecting survival were not observed.

**Conclusions:**

When comparing 3DCRT- versus IMRT-based dCRT, no survival benefits were observed. However, we found a lower local recurrence rate in the IMRT group potentially owing to dose-escalation. Prospective data are needed to verify the presented results herein.

## Background

Though the current standard of care for locally advanced esophageal cancer (EC) is neoadjuvant chemoradiotherapy (nCRT) followed by resection [1, 2], the latter is also associated with surgical morbidity and mortality. Two randomized trials have addressed nCRT versus definitive CRT (dCRT); despite local control (LC) benefits with surgery, no differences were discerned in distant metastasis or survival [3, 4]. Thus, dCRT is performed – and is indeed a viable recommendation – for select ECs [1, 5, 6].

However, virtually all seminal clinical trials for both nCRT and dCRT have utilized three-dimensional conformal radiotherapy (3DCRT) [2–4, 7, 8]. The advanced and highly conformal technology of intensity-modulated radiotherapy (IMRT) has heretofore been studied only in limited capacities [9–13]. It remains an attractive modality largely owing to the intimate anatomic relationship of the esophagus with both lungs and the heart. Indeed, several publications have posited a positive correlation between radiation dose to the lungs [14–18] and heart [19–22], with the risk of toxicities in these structures. Hence, the high conformality of IMRT could theoretically afford fewer toxicities to these organs-at-risk (OARs), especially in the setting of relative dose-escalation with dCRT, but supportive data are currently lacking. The aim of this study was to compare outcomes and acute toxicity of EC patients receiving definitive dCRT with either 3DCRT or IMRT.

## Methods

This single-institutional study was approved by the university institutional review board and ethics committee, and patient/treatment/outcomes were retrospectively collected from an institutional database. Herein, we evaluated 93 patients (2000–2012) treated definitively for locally advanced EC. Specifically, inclusion criteria were histologically-proven esophageal squamous cell or adenocarcinoma, Karnofsky performance status (KPS) ≥70, curative treatment intent, systemic workup negative for distant metastases, and receipt of concomitant CRT. Patients were excluded if receiving <50 Gy definitively, as per prior studies [2]. The choice of radiation modality (IMRT or 3DCRT) was made at the radiation oncologist’s discretion, predominantly influenced by institutional guidelines and availability at the time of treatment planning.

All patients underwent systemic workup with referring providers prior to commencing CRT and a CT for radiation treatment planning. The gross tumor was first contoured, followed by a 4–5 cm craniocaudal margin (0.5-1 cm radially) and inclusion of locoregional lymphatic pathways to form the clinical target volume (CTV); another isotropic margin 0.5-1 cm was added to construct the planning target volume (PTV). If a boost was applied, the boost CTV was defined by adding a margin of 1.5-2 cm craniocaudally and 0.5-1 cm radially. The boost CTV-to-PTV margin was 0.5-1 cm in any direction. 3DCRT utilized a 3-5-field beam arrangement. IMRT was planned using either a step-and-shoot design or helical application mode. Boosts were either sequential or simultaneous. Image guidance was utilized in more contemporary time periods.

Chemotherapy was administered concomitantly with RT in all patients, and consisted of cisplatin 20 mg/m^2^ + 5-FU 1000 mg/m^2^ over 5 days at weeks 1, 5, 9, and 13. While receiving CRT, patients were monitored weekly on treatment with toxicity assessments, and at regular follow-up intervals with imaging.

Statistical analyses were performed using R (version 3.0.2, R Development Core Team, 2013, URL: http://www.r-project.org/) combined with the packages ‘splines’, ‘survival’ (version 2.37-7, Therneau, 2014) and ‘xtable’ (version 1.7-1, Dahl, 2013). Tests were two-sided with *p* < 0.05 considered significant. Since this was an exploratory trial, all *p*-values are interpreted descriptively and no adjustment for multiple testing is applied. Chi-squared test assessed measures of association in frequency tables, and the t-test evaluated the equality of population distributions. Survival analysis was done using Kaplan-Meier methodology. A local relapse (LR) was defined as the locally recurrent disease including a biopsy-proven or radiologically-suspicious lesion in close proximity to the initial disease. Progression-free survival (PFS) was defined as the time from last oncologic treatment to death, local recurrence or distant metastasis, whichever occurred first. Overall survival (OS) referred to the time interval between initial diagnosis to death from any cause, with censorship based on particular follow-up times. The Cox proportional hazards model was used for univariate analysis (UVA) and multivariate analysis (MVA) to assess the effect of several clinical factors on LR, PFS and OS. The Wald test was used to assess the predictive value of the covariates in the model.

Toxicities were assigned at the time of initial occurrence by the treating physician according to the Common Terminology Criteria for Adverse Events v4.0, and retrospectively reviewed for purposes of this study.

## Results

Median and mean follow-up for all patients was 20.1 months and 34.7 months, respectively (range, 3 – 150.1 months). Table 1 displays clinical characteristics of the patient population. Of note, differences between groups included a more proximally localized upper tumor border (23.7 vs. 26.4 cm from the dental arcade, *p* = 0.031) and a higher mean cumulative radiation dose (57.2 vs. 55.4 Gy, *p* = 0.01) for the IMRT group.

**Table 1:** Characteristics of the patient cohort separated by radiotherapy technique

Characteristic	3DCRT	IMRT	*p*-value
	(*n* = 49)	(*n* = 44)	
Age – years			0.448
Mean	64.2	62.9	
Median	65	62	
Range	45-84	40-79	
Male Sex – no. (%)	42 (85.7)	36 (81.8%)	0.610
KPS – %			0.516
Mean	85.5	86.6	
Median	90	90	
Range	70-100	70-100	
T-Stage – no. (%)			0.331
T1	0 (0)	0 (0)	
T2	13 (26.5)	8 (18.2)	
T3	26 (53.1)	30 (68.2)	
T4	10 (20.4)	6 (13.2)	
N-Stage – no. (%)			0.507
N0	10 (20.4)	11 (25)	
N1	28 (57.1)	26 (59.1)	
N2	11 (22.4)	6 (13.6)	
N3	0 (0)	1 (2.3)	
Grade – no. (%)			0.367
G1	1 (2)	2 (4.5)	
G2	20 (40.8)	23 (52.3)	
G3	28 (57.1)	19 (43.2)	
Pretreatment Hemoglobin – g/dl			0.394
Mean	12.8	13.1	
Median	12.6	13.5	
Range	9.5-15.9	5.2-17.1	
Location (Distance from dental arcade to cranial tumor border) – cm			0.031
Mean	26.4	23.7	
Median	27	22	
Range	15-37	10-38	
Histology – no. (%)			0.491
Squamous cell	41 (83.7)	39 (88.6)	
Adeno	8 (16.3)	5 (11.4)	
Cumulative RT dose – Gy			0.010
Mean	55.4	57.2	
Median	54	56.8	
Range	50.4-70.2	50–61.2	

**Fig. 1:**
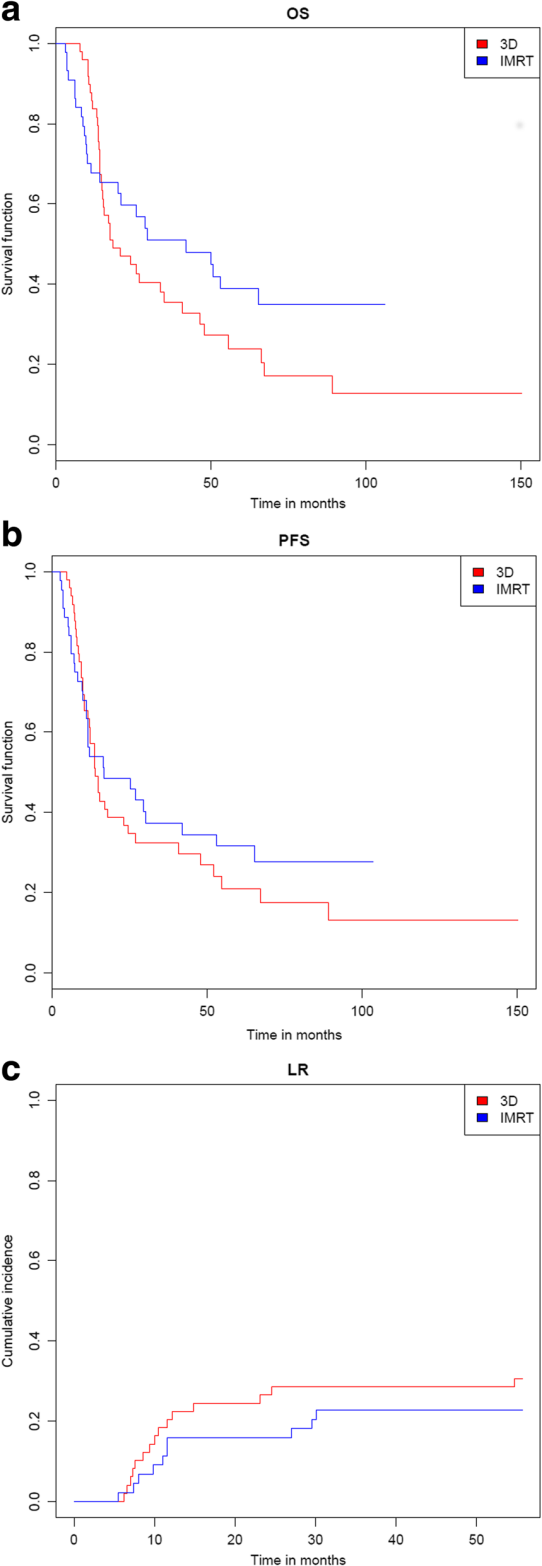
Kaplan-Meier estimates of **a** OS, **b** PFS and **c** LR for both groups

Figure 1 illustrates results of local relapse rate (LR), OS and PFS for both groups. The 1- and 3-year LR was 20.4% and 28.6% in the 3DCRT group and 15.9% and 22.7% in the IMRT group, respectively (*p* = 0.620). Median PFS in the respective groups were 13.8 and 16.6 months, which corresponded to 3- and 5-year PFS of 32.4% and 21% compared to 37.4% and 31.6%, respectively (*p* = 0.448). Median OS in the respective cohorts were 18.4 and 42.0 months, which corresponded to 3- and 5-year OS of 35.5% and 23.9% compared to 50.9% and 38.9%, respectively (*p* = 0.198).

Univariate analysis (UVA) determined factors associated with LR, PFS, and OS (Table 2). None of the investigated factors influenced OS or PFS with statistically significant impact. However, there was a trend for worse OS and PFS for high-grade disease (*p* = 0.052 and *p* = 0.089, respectively) and for improved PFS with higher pre-treatment hemoglobin levels (*p* = 0.057). Higher cumulative RT doses beneficially impacted LR on UVA (*p* = 0.004). Multivariate analysis (MVA) included the factors RT technique, KPS, T-stage, N-stage, Grading, tumor histopathology and RT dose. Besides the confirmation of a beneficial impact of increased RT doses on LR (HR 0.686, 95%-CI 0.524 – 0.897; *p* = 0.006) and a borderline significant worse OS for high-grade tumors (HR 1.743, 95%-CI 1.003 – 3.029; *p* = 0.049) MVA did not reveal any further significant findings.

**Table 2:** Hazard ratios (HR) with 95%-confidence intervals (CI) in univariate analysis for OS, PFS and LR

	OS		PFS		LR	
*Parameter*	HR [95% CI]	*p*-value	HR [95% CI]	*p*-value	HR [95% CI]	*p*-value
RT mode (IMRT vs. 3DCRT)	0.716 [0.430; 1.191]	0.198	0.830 [0.512; 1.344]	0.448	0.812 [0.356; 1.852]	0.620
Age (continuous)	1.006 [0.978; 1.035]	0.676	1.009 [0.981; 1.037]	0.525	0.962 [0.906; 1.022]	0.211
KPS (continuous)	1.004 [0.973; 1.035]	0.816	1.024 [0.992; 1.057]	0.138	0.988 [0.940; 1.039]	0.644
T-stage (T3/4 vs. T1/2)	1.177 [0.637; 2.178]	0.603	1.226 [0.679; 2.212]	0.499	1.170 [0.411; 3.328]	0.768
N-stage (N1-3 vs. N0)	1.493 [0.779; 2.863]	0.228	1.399 [0.764; 2.561]	0.277	1.218 [0.408; 3.635]	0.723
Grade (G3 vs. G1/2)	1.651 [0.996; 2.737]	0.052	1.518 [0.938; 2.456]	0.089	1.040 [0.455; 2.374]	0.926
Pretreatment Hemoglobin	0.930 [0.821; 1.054]	0.255	0.886 [0.783; 1.003]	0.057	0.975 [0.821; 1.157]	0.768
Location	1.002 [0.960; 1.046]	0.935	0.992 [0.952; 1.033]	0.689	1.030 [0.958; 1.107]	0.422
Histology (Adeno vs. SCC)	1.555 [0.786; 3.076]	0.205	1.630 [0.851; 3.125]	0.141	1.354 [0.489; 3.750]	0.560
RT dose (continuous)	0.946 [0.881; 1.015]	0.122	0.955 [0.893; 1.021]	0.173	0.763 [0.635; 0.916]	0.004
Treatment year (continuous)	1.021 [0.934; 1.116]	0.646	1.043 [0.958; 1.135]	0.329	1.041 [0.893; 1.213]	0.606

Acute, radiation-related toxicities experienced by both cohorts are displayed in Table 3. These predominantly included dysphagia, radiodermatitis and nausea/vomiting. Of note, dysphagia was observed in 70.5% for patients in the IMRT group vs. 42.9% in the 3DCRT group (*p* = 0.067). Owing to a short follow-up period and assumed issues with data integrity regarding late toxicity reporting in the cohorts, late toxicities were unable to be reliably presented herein.

**Table 3:** Acute toxicities related to radiotherapy in the groups of 3DCRT and IMRT

	3DCRT	IMRT	*p*-value
	(*n* = 49)	(*n* = 44)	
Dysphagia – no. (%)			0.067
absolute	21 (42.9)	31 (70.5)	
G1	4 (8.2)	9 (20.5)	
G2	15 (30.6)	15 (34.1)	
G3	2 (4.1)	6 (13.6)	
G4	0	1 (2.3)	
Radiodermatitis – no. (%)			0.638
absolute	14 (28.6)	17 (38.6)	
G1	9 (18.3)	13 (29.6)	
G2	3 (6.1)	2 (4.6)	
G3	2 (4.1)	2 (4.6)	
G4	0	0	
Nausea/Vomiting – no. (%)			0.602
absolute	11 (22.4)	9 (20.5)	
G1	8 (16.3)	4 (9.1)	
G2	2 (4.1)	4 (9.1)	
G3	1 (2.0)	1 (2.3)	
G4	0	0	
Mucositis – no. (%)			0.518
absolute	7 (14.3)	7 (15.9)	
G1	2 (4.1)	0	
G2	4 (8.2)	4 (9.1)	
G3	1 (2.0)	3 (6.8)	
G4	0	0	
Bleeding – no. (%)			0.341
absolute	1 (2.0)	0	
G1	1 (2.0)	0	
G2	0	0	
G3	0	0	
G4	0	0	
Pneumonitis – no. (%)			0.557
absolute	3 (6.1)	1 (2.3)	
G1	1 (2.0)	0	
G2	2 (4.1)	1 (2.3)	
G3	0	0	
G4	0	0	

In terms of dosimetric analysis, detailed treatment plan information was available in 76 patients. Basic dosimetric parameters of 3DCRT and IMRT plans are shown in Table 4. A trend towards larger PTVs was observed for IMRT (*p* = 0.060). There were no significant differences in normal tissue exposure between treatment techniques.

**Table 4:** Dosimetric parameters related to radiotherapy in the groups of 3DCRT and IMRT

	3DCRT	IMRT	*p*-value
	(*n* = 39)	(*n* = 37)	
Boost concept – no. (%)			<0.001
sequential	36 (92.3)	16 (43.2)	
integrated	0 (0)	16 (43.2)	
none	3 (7.7)	5 (13.5)	
PTV volume - ml			0.060
Mean	667	796	
Median	657	755	
Range	229-1236	193-1715	
Boost PTV volume - ml			0.139
Mean	236	192	
Median	230	175	
Range	73-528	24-624	
Mean lung dose - Gy			0.651
Mean	12.6	12.2	
Median	12.3	12.4	
Range	4.8-20.2	3.1-18.9	
Mean heart dose - Gy			0.158
Mean	18.1	14.2	
Median	19.7	14.5	
Range	0.4-38.8	0.2-37.5	
Spinal cord D_max_ - Gy			0.229
Mean	36.8	35.1	
Median	38	36.3	
Range	14.2-45.9	8–43.2	

## Discussion

In our study cohort, the choice of radiation technique (3DCRT or IMRT) did not significantly influence survival in definitive chemoradiotherapy for esophageal cancer patients. These results correspond to previous findings [9, 10], yet contradictory results have been presented as well [13]. Nevertheless, the value of such an analysis is not diminished, because there is still a lack of data regarding the potential value of IMRT in the definitive, relatively dose-escalated setting. This will undoubtedly be an important issue in the coming years as the use of IMRT for EC increases [23].

The novelty and emphasis of our data were aimed at detecting differences in outcome endpoints in the setting of relative dose-escalation (in the definitive setting). Although median OS was 42.0 months with IMRT compared to 18.4 months with 3DCRT, this difference did not reach statistical significance. However, LR was significantly increased by higher cumulative radiation doses. Interestingly, radiation modality did not show a noticeable impact on LR though confounder analysis revealed a significantly higher mean cumulative RT dose in the IMRT group. We suppose that this difference between both groups, namely 1.8 Gy (3DCRT 55.4 Gy vs. IMRT 57.2 Gy, *p* = 0.01), is too small to translate into a considerable outcome difference of radiation techniques. However, our data reveal a dose-relationship resulting in an increased local tumor control with higher radiation doses. This finding is in line with other investigations that have noted outcome enhancements for dose-escalation [24]. In contrast, the RTOG 9405 trial did not discern advantages from dose-escalation [25], albeit with now-outdated techniques. In summary, our data support the hypothesis that increased radiation doses improve patient outcomes; however, the potential benefit of dose-escalation in EC remains inconclusive due to the heterogeneity of evidence [26].

Nevertheless, there is little debate that IMRT theoretically allows for safer dose-escalation. Dosimetric investigations have determined that advanced IMRT techniques provide numerical advantages over 3DCRT, but without outcome differences [27, 28]. This particularly applies to a reduction of high dose exposure to the OAR. For example, Münch et al. have demonstrated a significant reduction of the lung V_30Gy_ with VMAT (6.6%) compared to 3DCRT (11%) and of the heart V_30Gy_ (17.7% with VMAT vs. 50.4% with 3DCRT) [27]. In contrast, our study failed to demonstrate any significant differences in OAR dose exposure. However, slightly lower doses to the spinal cord and the heart were observed for IMRT and MLD was equal despite a larger PTV volume by trend of IMRT patients. Of note, the nature of IMRT and arc therapy results in increased low-dose areas to the heart and lungs, the ramifications of which are also unclear presently. In the study of Münch et al., the V_5Gy_ of the lungs and the heart were 90.1% and 100% with VMAT compared to 79.7% and 91% with 3DCRT, respectively [27]. Using rotational techniques, low-dose areas to the lung have been higher in some studies than the recommended limits in other (lung cancer) studies [13, 29]. It cannot go unmentioned, however, that inverse-planned therapies (e.g. IMRT, arc therapy, etc.) are heavily dependent on nuances of treatment planning, technique, delivery, and optimization, which can lead to a variety of potential results [30]. The use of helical IMRT herein has heretofore never been reported as a modality of IMRT delivery, and could be of further interest for future investigations.

Though there is a knowledge gap regarding the role of IMRT in EC, compelling data suggest that IMRT and other advanced techniques may decrease postoperative complications when used in the setting of nCRT, thus potentially decreasing surgical morbidity and mortality [17]. Notably, Lin et al. analyzed multiple cancer registries and performed inverse probability of treatment weighting-adjusted multivariate analysis [31]. Therein, radiation modality (3DCRT vs. IMRT) was independently associated with all-cause, other, and cardiac-specific mortality. It is thus important to be cognizant of cardiac doses in a neoplasm for which the heart was historically not a major priority but long-term survival is now increasing; this is similar to the findings of the RTOG 0617 trial in locally advanced non-small cell lung cancer (NSCLC) [32]. Because IMRT affords lower cardiac doses for both tumors, it has been postulated that IMRT should be the standard for NSCLC, despite a similar lack of comparative data in both NSCLC and EC [33].

There are several limitations of our study. In addition to the retrospective nature and smaller sample size, it is recognized that groups were somewhat imbalanced in terms of tumor location. The rather short follow-up time may also likely underestimate cardiac events, as these typically take at least 5–10 years to develop. Although the year of treatment was not a significant factor on outcome in UVA, advancements in diagnostics and treatment options after the first line during the inclusion period of twelve years may cause an undiscovered bias. Other newer supportive technologies also cannot be overlooked, such as staging PET and 4-dimensional CT planning. Moreover, patients with poor KPS were excluded, which also contribute a nontrivial proportion of EC patients. Also, limiting applicability were the low numbers of adenocarcinoma, as well as institutional/regional biases in referring patients for surgery versus definitive therapy. Taken together, however, our data should ideally signal other reports as to whether IMRT is indeed appropriate for several patient subgroups over 3DCRT.

If clinical benefits of IMRT in the definitive and/or neoadjuvant treatment of EC are eventually proven, it could signal further testing of an even more new modality known as proton beam therapy. Though issues with economic costs remain, this modality has been also associated with low perioperative complications, which could potentially offset these costs [34–36]. A randomized trial of photons versus protons for EC is currently underway (NCT01512589).

## Conclusions

These data showed that when comparing 3DCRT and IMRT, survival of patients with esophageal cancer is not affected. However, we found a lower rate of local relapses in the IMRT group potentially owing to dose-escalation. Further validation is needed from prospective studies.

## Abbreviations

3DCRT: 3D conformal radiotherapy
CI: Confidence interval
CRT (dCRT/nCRT): Chemoradiotherapy (definitive/neoadjuvant)
CTV: Clinical target volume
D_max_: Maximum dose
EC: Esophageal cancer
HR: Hazard ratio
IMRT: Intensity-modulated radiotherapy
LR: Local relapse
MLD: Mean lung dose
MVA: Multivariate analysis
NSCLC: Non-small cell lung cancer
OAR: Organ(s) at risk
OS: Overall survival
PET: Positron emission tomography
PFS: Progression-free survival
PTV: Planning target volume
RTOG: Radiation Therapy Oncology Group
UVA: Univariate analysis
VMAT: Volumetric Arc Therapy
